# Recent Mortality from Pleural Mesothelioma, Historical Patterns of Asbestos Use, and Adoption of Bans: A Global Assessment

**DOI:** 10.1289/ehp.11272

**Published:** 2008-08-14

**Authors:** Kunihito Nishikawa, Ken Takahashi, Antti Karjalainen, Chi-Pang Wen, Sugio Furuya, Tsutomu Hoshuyama, Miwako Todoroki, Yoshifumi Kiyomoto, Donald Wilson, Toshiaki Higashi, Megu Ohtaki, Guowei Pan, Gregory Wagner

**Affiliations:** 1 Department of Environmental Epidemiology, Institute of Industrial Ecological Sciences, University of Occupational and Environmental Health, Kitakyushu City, Japan; 2 Finnish Institute of Occupational Health, Helsinki, Finland; 3 Centre for Health Policy Research and Development, National Health Research Institutes, Taiwan; 4 Japan Occupational Safety and Health Resource Centre, Tokyo, Japan; 5 Department of Work, Systems, and Health, Institute of Industrial Ecological Sciences, University of Occupational and Environmental Health, Kitakyushu City, Japan; 6 Department of Environmetrics and Biometrics, Hiroshima University, Hiroshima, Japan; 7 Department of Environmental Epidemiology, Liaoning Provincial Centre for Disease Prevention and Control, Shenyang, People’s Republic of China; 8 U.S. National Institute for Occupational Safety and Health, Washington, DC, USA

**Keywords:** asbestos, asbestos-related diseases, ban, epidemiology, lung cancer, mesothelioma, mortality, occupational cancer, pleural mesothelioma

## Abstract

**Background:**

In response to the health risks posed by asbestos exposure, some countries have imposed strict regulations and adopted bans, whereas other countries have intervened less and continue to use varying quantities of asbestos.

**Objectives:**

This study was designed to assess, on a global scale, national experiences of recent mortality from pleural mesothelioma, historical trends in asbestos use, adoption of bans, and their possible interrelationships.

**Methods:**

For 31 countries with available data, we analyzed recent pleural mesothelioma (*International Classification of Diseases, 10th Revision*) mortality rates (MRs) using age-adjusted period MRs (deaths/million/year) from 1996 to 2005. We calculated annual percent changes (APCs) in age-adjusted MRs to characterize trends during the period. We characterized historical patterns of asbestos use by per capita asbestos use (kilograms per capita/year) and the status of national bans.

**Results:**

Period MRs increased with statistical significance in five countries, with marginal significance in two countries, and were equivocal in 24 countries (five countries in Northern and Western Europe recorded negative APC values). Countries adopting asbestos bans reduced use rates about twice as fast as those not adopting bans. Turning points in use preceded bans. Change in asbestos use during 1970–1985 was a significant predictor of APC in mortality for pleural mesothelioma, with an adjusted *R*^2^ value of 0.47 (*p* < 0.0001).

**Conclusions:**

The observed disparities in global mesothelioma trends likely relate to country-to-country disparities in asbestos use trends.

The world is steadily retreating from dependence on asbestos. In 2006 the International Labour Organization (ILO 2006) and World Health Organization (WHO 2006a) jointly declared that the most efficient way to eliminate asbestos-related diseases is to stop using all types of asbestos. Nevertheless, current use varies widely. Some countries have imposed strict regulations to limit exposure, others have adopted bans, and yet others have intervened less and have continued to use varying quantities of asbestos. The global burden of asbestos diseases over time will be uneven, reflecting the extent and patterns of asbestos use.

Globally, each year, an estimated 125 million people are occupationally exposed to asbestos, and 90,000 die from asbestos diseases (WHO 2006a). Around the time of peak use in the mid-1970s, approximately 25 countries produced asbestos and 85 countries manufactured asbestos products (Virta 2005). In 1983, Iceland became the first country to ban asbestos, reflecting increasing recognition, predominantly in Western countries, of health risks associated with asbestos exposure. Subsequently, 40 or more countries have adopted bans (WHO 2006a).

Among the asbestos diseases, mesothelioma is the most sensitive and specific indicator of the disease burden in the population (Weill et al. 2004). The annual incidence of mesothelioma has been estimated at 10,000 cases in Western Europe, North America, Japan, and Australia combined (Anonymous 1997). Peto et al. (1995, 1999) predicted a dramatic increase in future mesothelioma deaths in the United Kingdom and Europe. Several statistical projections have been made since then, suggesting that deaths from mesothelioma will increase in many countries.

We recently reported that per capita asbestos use is a useful surrogate for the general asbestos exposure level of a population and may be used for estimation of health effects (Lin et al. 2007). Information is limited at the global level concerning the relationship between mesothelioma trends and trends in asbestos use, and the status of bans. Our aim in the present study was to assess, on a global scale, national experiences of recent mortality from mesothelioma, historical trends in asbestos use, adoption of bans, and their possible interrelationships. We focused specifically on pleural mesothelioma in men because a high proportion of such cases arise from asbestos exposure.

## Materials and Methods

### Indicators of mortality

The primary source of information on mortality was the WHO database (WHO 2006b). It registers the number of deaths by country according to the *International Classification of Diseases* (ICD). Several countries shifted from coding based on the ICD *9th Revision* (ICD-9) to that based on the *10th Revision* (ICD-10) (WHO 1992) during our 1996–2005 study period [year of change ranged from 1996 to 2002, with a median of 1998 in the countries studied; Supplemental Material, Table 1 (http://www.ehponline.org/members/2008/11272/suppl.pdf)]. Notably, the disease category of mesothelioma was initially introduced into ICD-10 codes comprising subcategories of pleural (C45.0), peritoneal (C45.1), pericardial (C45.2), other sites (C45.7), and unspecified (C45.9). In our study, we defined pleural mesothelioma as a composite of mesothelioma of the pleura (C45.0) and unspecified mesothelioma (C45.9) because in certain countries, including the United States, most mesothelioma was coded as C45.9 instead of C45.0. From the database, we obtained the annual numbers of male deaths for each country, based on 5-year age intervals.

We obtained national population data from the WHO (2006b), the U.S. Census Bureau (2006), the United Nations (2006), and Lahmeyer (2007), prioritized for use in that order. For each country, we calculated age-adjusted annual mortality rates (annual MRs; deaths/million/year) by dividing the number of male deaths in each year by the size of the corresponding male national population, which we age-standardized to the world standard population of the year 2000 (Ahmad et al. 2000). We similarly calculated period MRs by dividing the average annual number of male deaths from 1996 to 2005 by the average sizes of male national populations, also age-standardized.

To characterize the trend of mortality, we estimated the annual percent change (APC) of annual MRs using the Joinpoint software (version 3.0, U.S. National Cancer Institute, Bethesda, MD, USA). Briefly, the method fits a least-squares regression line to the natural logarithm of the rates using calendar year as a regressor variable. That is, *y* = *bx* + *c*, where *y* is the ln(rate), *x* is the calendar year, and *c* is the intercept. Hence, APC = 100 × (*e^b^* – 1) (Jemal et al. 2000; Lasithiotakis et al. 2006; Ries et al. 1997). In addition, we calculated *p*-values for APC = 0 and 95% confidence intervals (CIs) of APCs. Testing the hypothesis that APC = 0 is equivalent to testing the hypothesis that the regression slope parameter is equal to zero (Ries et al. 1997). We assumed a linear change of trends in log rates over time. Because trends pertained to a 10-year period, we limited analyses to countries with at least 4 years of pleural mesothelioma data under ICD-10 codes (the range was 4–9 years, with a median of 6 years).

### Indicators of asbestos use

We extracted data on new use of asbestos by country from a U.S. Geological Survey (USGS) report (Virta 2006). We defined “use” as production plus import minus export (Virta 2006). We considered negative values of use (caused by storage and the like) uninformative and excluded them from further analyses. To characterize trends, we divided use numbers by sizes of national populations for the corresponding year or period (to give use per capita, expressed as kilograms per capita/year) (Lin et al. 2007). The USGS database provides data only sparsely in 10-year intervals up to 1960, 5-year intervals from 1970–1995, and annually for 1996–2003. We classified use of ≥ 3.0 kg per capita/year as high and ≥ 4.0 as very high, and change in use during a particular period (Δ, kilograms per capita/year) as the difference between average use during the earlier and latter subperiods (halves) of the entire period (e.g., for the period 1960–1985, change is the difference between the average use of 1960 and 1970 and the average use of 1975 and the average use of 1980, and 1985; for the period 1970–1985, change is the difference between the average use of 1970 and 1975 and the average use of 1980 and 1985). We calculated Δ values for all possible combinations of available data. We retrieved national ban status from the database compiled by Kazan-Allen (2005, 2006) and verified it by separate reports. To describe historical trends in asbestos use and relationships with banning status, we grouped countries according to their national ban status into early-ban (adopted by 1995), late-ban (1996–2006), and no-ban groups.

### Statistical analysis

We adapted geographic grouping of countries from the U.N. Statistics Division (United Nations 2006). We performed statistical analyses using Joinpoint, SPSS version 12.0 (SPSS Inc., Chicago, IL, USA), and Excel 2003 (Microsoft Corp., Redmond, WA, USA). When we used Joinpoint, we assumed a linear change (or 0 joinpoint) during the observed period, with a maximum length of 10 years. We deemed *p* < 0.05 statistically significant and 0.05 < *p* < 0.10 marginally significant. We use the terms “increase” (denoted as ↑) or “decrease” (↓) when APC was marginally or statistically significant, and “equivocal” (↔) when APC and its significance level were neither statistically nor marginally significant.

When we evaluated trends in asbestos use by groups of countries, we weighted means by the size of national populations of the corresponding periods. We analyzed data from the United States separately because of the known high degree of historical asbestos use. We regressed recent changes in pleural mesothelioma mortality (APC values) against historical changes in use of asbestos (Δ values for various periods). We weighted each regression model by the sizes of male national populations in the corresponding period.

## Results

### Trends in mortality

Table 1 shows the period MRs and APCs in mortality for pleural mesothelioma and male population by country. Mortality from pleural mesothelioma was highest in United Kingdom (31.1 deaths/million/year), with a global median of 7.8 deaths/million/year. Trends of mortality were as follows: statistically significant increases in five countries, marginally significant increases in two countries, and equivocal results in 24 countries. Global median APC was 4.5%/year, and negative values of APC were recorded in five countries of Northern and Western Europe. We observed increasing trends more often in countries with above-median period MR values than in those with below-median values (26.7%, or 4 of 15, vs. 20.0%, or 3 of 15).

Regionally, countries of Northern and Western Europe and Oceania showed high and stable MRs; those of Eastern and Southern Europe, South America, and Asia showed low and increasing rates.

### Trends in asbestos use

Asbestos use peaks were higher and occurred earlier in the countries of Northern and Western Europe, Oceania, and the Americas (excluding South America) (Table 2). Very high (≥ 4.0 kg per capita/year) asbestos use was recorded in Australia, Canada, and several countries of Northern and Western Europe.

**Figure 1:**
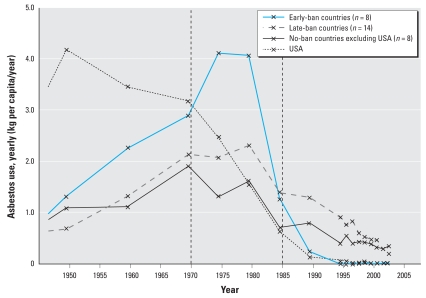
Historical trends in use of asbestos from 1950 to 2003 grouped by status of national bans. Early-ban countries are countries that adopted bans in 1995 or before (*n* = 8); late-ban countries adopted bans from 1996 to 2006 (*n* = 14); no-ban countries, excluding the United States, did not adopt bans until 2007 (*n* = 8). Asbestos use (*y*-axis) is per capita yearly use (averages weighted by the sizes of national populations). The USGS (Virta 2006) database provides data only sparsely in 10-year intervals up to 1960, 5-year intervals from 1970–1995, and annually for 1996–2003. Straight lines connect available data.

Asbestos use fell most quickly in countries that adopted early bans, at an intermediate rate in countries with late ban adoption, and most slowly in countries without bans (Figure 1). Specifically, the early-ban group, during its period of adopting bans, recorded a reduction rate of −8.3%/year, from 2.4 kg per capita/year in 1983 (first ban) to < 0.01 kg per capita/year in 1995 (last ban). This was about twice as fast as the late-ban and no-ban groups, which recorded a reduction rate of −4.1%/year and −5.2%/year, respectively, during the same period. Similarly, the late-ban group, during its period of adopting bans, recorded a reduction rate of −10.7%/year, from 0.7 kg per capita/year in 1996 (first ban) to 0.2 kg per capita/year in 2003. During the same period, the value for the no-ban group was −4.9%/year, resulting in a 2.2-fold quicker reduction rate in the late-ban group. The historical use pattern of the United States differed from that of other countries. The United States recorded the earliest and maximal peak use at 4.2 kg per capita/year in 1950, followed by progressive reduction over four decades and approaching 0.02 kg per capita/year in 2003, equating to a reduction rate of −1.9%/year. The no-ban group had the lowest peak but currently maintains the highest level of asbestos use at 0.4 kg per capita/year. The period of 1970–1985 contained historical use peaks with a notable shift to downward trends for many but not all countries.

### Interrelationships

**Figure 2:**
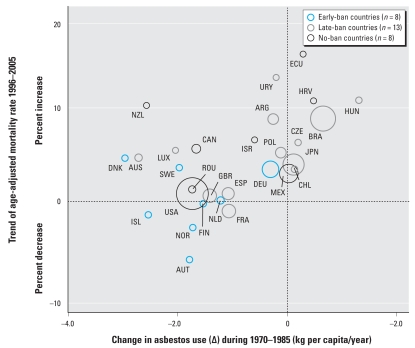
Trend of MRs for male pleural mesothelioma in relation to change in asbestos use. See Table 1 for country codes. Circles have areas proportional to the sizes of male national populations; the smaller equal sizes indicate male national populations < 5,000,000. We defined the trend of MRs (*y*-axis) as APC, as calculated by the Joinpoint software. Bivariate relationships were examined by linear regression, weighted by the sizes of male national populations, and produced the following model: *y* = 0.011*x* + 2.022 (adjusted *R*^2^ = 0.47, *p* < 0.0001).

The change in asbestos use (Δ) during 1970–1985 was the strongest predictor of APC among the many periods tried, with an adjusted *R*^2^ value of 0.47 (*p* < 0.0001) (Table 3). Changes in asbestos use during other adjacent periods (e.g., 1960–1990–1970–1990) also predicted APC in mortality, each with relatively high statistical significance. Figure 2 shows the positive log-linear relationships between changes in asbestos use and APCs in mortality, where increments in recent MRs are associated with increments in historical asbestos use.

## Discussion

The present study identified wide differences in recent mortality from pleural mesothelioma in various countries. Recent MRs were highest in the countries of Northern and Western Europe and Oceania. Increasing trends, as measured by APCs in mortality, were common in the countries of Eastern and Southern Europe, Asia, and South America.

We assessed mortality trends over the most recent 10-year window, using the earliest opportunity to analyze the disease under the standard code of ICD-10. However, the study period was inadequate to depict trends in many countries. National data recorded only under ICD-9 had to be precluded (e.g., Italy). For the countries shifting from ICD-9 to ICD-10 during the study period, we limited our analyses to the period when data were recorded under ICD-10.

Further, data may lack comparability, especially because mesothelioma is rare and difficult to diagnose. A major concern is that increasing trends recorded in countries with low mortality levels could be explained by improved disease recognition (Peto et al. 1995; Weill et al. 2004), and such secular trends in diagnosis would be statistically indistinguishable from real increases (Peto et al. 1995). Our study revealed increasing mortality trends in the group that recorded above-median values for the period MR (group 1) than the group that recorded below-median values for the period MR (group 2). Such bias is likely to be less serious in group 1 than group 2. Thus, although increases in disease recognition are probable, this factor alone does not explain the increasing trends. The proportionality with which recent mortality trends were related to historical trends of asbestos use offers a more compelling explanation.

Pleural mesothelioma is the predominant type of mesothelioma and is strongly related to asbestos exposure. However, in certain countries, most mesothelioma was coded into the subcategory of unspecified mesothelioma (C45.9) instead of the subcategory of pleural mesothelioma (C45.0): the ratio of C45.0 to C45.0 + C45.9 ranged from 0.08 (Israel), 0.11 (United States), and 0.12 (Canada) to 0.94 (New Zealand) and 0.98 (Finland), with a median of 0.63. We therefore created a composite category of C45.0 and C45.9 to ensure comparability, which we deemed more reasonable than the alternative choices of analyzing only C45.0 or mixing C45.0 with other subcategories—for example, peritoneal (C45.1) or pericardial (C45.2) or other sites (C45.7).

Our findings on mortality trends are comparable with trends reported earlier for individual countries, including the Netherlands (Segura et al. 2003), Sweden (Burdorf et al. 2005), Finland (Karjalainen et al. 1997), and Denmark (Kjaergaard and Andersson 2000), as well as overall Europe (Montanaro et al. 2003). However, methods and indices employed to evaluate trends are unique to each study, and comparisons cannot exceed the general trend characteristics. For the United States, we recorded equivocal trends (i.e., APC = 0.8%). Similarly, Price (1997) first observed that the annual growth rate during 1973–1992 was declining, and Price and Ware (2004) reported “no substantive changes in time pattern of mesothelioma incidence since 1992.” Furthermore, surveillance information in United States does not show an apparent trend from 1999 to 2002 (National Institute for Occupational Safety and Health 2005).

Regarding historical trends in asbestos use, we identified several distinctive patterns: *a*) a very early (1950) and very high (≥ 4.0 kg per capita/year) peak followed by a progressive decline (in the United States); *b*) a mid-term (1960s–1980s) very high peak, followed by an abrupt decline (Australia and several Northern and Western European countries); and *c*) a late (≥ 1980) and relatively moderate peak followed by a moderate decline (Hungary and Japan).

In the United States, a “bubble” in asbestos use occurred in the mid-20th century because of early manufacturing research, industrial demand, and ready supply from Canada (Virta 2006). However, the United States was also the first to experience the burst of the bubble due to growing health concerns and liability issues (Virta 2006). In 1989, the U.S. Environmental Protection Agency (EPA) banned most asbestos-containing products, but this regulation was overturned by the U.S. Court of Appeals in 1991 (U.S. EPA 1989). Nevertheless, use fell to 4,600 tons in 2003 (0.7% of peak use). In many other countries, increasing use of asbestos paralleled the growth curves of industrialization.

Generally, countries recording early and high levels of asbestos use displayed peaks by 1980 followed by downward trends. The turning points preceded the earliest bans and are thus not direct outcomes of bans. Rather, paths leading to bans likely entailed regulatory restrictions and economic incentives and disincentives, which furthered reduction of use. Virta (2005, 2006) attributed maturation of the asbestos market superimposed on health issues as the main reason for the decline in use since 1980. Several relevant events with international impact coincided with this period. The International Agency for Research on Cancer (IARC), after acknowledging the carcinogenicity of asbestos in 1973 (IARC 1973), classified asbestos as a human carcinogen in 1977 (IARC 1977). The ILO added lung cancer and mesothelioma caused by asbestos to its list of occupational diseases in 1980 (ILO 1980) and adopted the Asbestos Convention in 1986 (ILO 1986). It was also around this period that the landmark studies by Selikoff and colleagues (Nicholson et al. 1982; Selikoff et al. 1984a, 1984b) gained wide recognition.

The adoption of bans by Northern European countries in the 1980s set a precedent for other countries, but the particular restrictions imposed by a “ban” vary by country, and the rates at which the absolute zero use levels were reached also vary. Collectively, countries adopting bans reduced use about twice as fast as those with lesser interventions. Notably, the countries of Eastern and Southern Europe (grouped here as “other” countries in Table 2) have continued to use asbestos, approaching high levels even after the turn of the century. The recent per capita use for the “other” Asian countries is low but shows little sign of decreasing. This is largely attributable to sustained use in China and India. Hence, our findings reinforce the widely held concern that the center of asbestos use is shifting to industrializing countries (Kazan-Allen 2005; LaDou 2004; Takahashi and Karjalainen 2003). Moreover, if the ecologic relationship reported here holds true for the future, corresponding risks should be anticipated in these countries.

Regression analyses showed the strongest relationship between recent APC in mortality from pleural mesothelioma and change in asbestos use during 1970–1985 (adjusted *R*^2^ = 0.47, *p* < 0.0001). The same analyses incorporating countries with six or more data points produced similar results (data not shown). The strong relationship is largely attributable to countries recording recent mortality trends in the same direction as historical use trends (lower-left and upper-right quadrants in Figure 2). The positive correlations found for change indicators of a number of periods in the present study reinforce the notion that per capita asbestos use is related to subsequent mortality level at the national level, as we reported earlier using absolute-level indicators (Lin et al. 2007). However, the time difference (i.e., latency) for the best predictive model was only 22.5 years (from mid-1977 to 2000), and thus the observed relationship may have reflected only early effects. In this connection, recent mortality trends of the eight early-ban countries are noteworthy: Seven countries recorded had equivocal MR trends, and only Germany had an increase in MR trend (Table 1). Germany actually recorded a historical use peak in 1980, trailing other early-ban countries by 5–10 years (detailed data not shown) and presumably delaying favorable changes in mortality trend. Continuing use of asbestos results in the accumulation of asbestos in the environment, thus creating possibilities for ongoing exposure due to maintenance, repair, and demolition during the entire life span of asbestos products. Given the long latency time, the mortality data available did not allow us to analyze the full consequences of such effects after the new use in longer term. Nevertheless, we observed significant (albeit weaker) relationships for changes in use during other close periods with longer latencies [e.g., 1950–1985 (latency 32.5 years) and 1950–1990 (30 years)].

In this study, we took advantage of the earliest opportunity to analyze mortality trends in a range of countries. Limitations included our dependence on a crude indicator of exposure (i.e., asbestos use per capita for sparse years with limited data), “bans” entailing varying restrictions on use that could not be measured, and no distinctions available between asbestos fiber types. Mortality data were limited to 31 countries, with developing countries likely lacking well-developed surveillance systems to assure quality of data. Moreover, the observed relationships are ecologic at the national level only, so all findings should be cautiously interpreted.

Because there is no safe threshold of exposure to asbestos, any degree of contact will involve some risk. On the other hand, the degree of risk is related to exposure. The experience of many countries suggests that attempts to reduce exposure without a concurrent reduction in overall use are insufficient to control risk. Countries implementing bans recorded reductions in asbestos use about twice as fast as those not adopting bans, for which our study period was probably too early to observe their full effects. However, the observed disparities in global mesothelioma trends are likely to relate to country-to-country disparities in asbestos use trends.

## Figures and Tables

**Table 1:** Recent trend in mortality from pleural mesothelioma^a^ in men.

Country (code)	Period MR^b^ (no.^c^) (deaths/million/year)	APC^d^ [%/year (95% CI)]	Trend^e^	Male population^f^ (million)
Asia				
Israel (ISR)	5.5 (5)	6.6 (−14.9 to 33.4)	↔	3.1
Japan (JPN)	4.8 (9)	3.9 (2.6 to 5.2)	↑**	61.4
Eastern Europe and Southern Europe				
Croatia (HRV)	8.8 (9)	11.0 (2.7 to 20.0)	↑**	2.2
Czech Republic (CZE)	3.2 (9)	6.3 (−1.7 to 15.0)	↔	5.0
Hungary (HUN)	2.5 (8)	11.0 (3.3 to 19.3)	↑**	4.9
Poland (POL)	2.0 (6)	5.2 (−5.2 to 16.7)	↔	18.7
Romania (ROU)	1.9 (6)	1.2 (−11.2 to 15.3)	↔	10.9
Spain (ESP)	5.7 (6)	0.7 (−6.6 to 8.7)	↔	19.8
Northern Europe and Western Europe				
Austria (AUT)	7.8 (4)	−5.9 (−20.9 to 12.0)	↔	3.9
Denmark (DNK)	12.9 (6)	4.6 (−6.5 to 16.9)	↔	2.6
Finland (FIN)	12.6 (9)	−0.3 (−3.9 to 3.6)	↔	2.5
France (FRA)	12.7 (4)	−1.0 (−14.7 to 14.9)	↔	28.7
Germany (DEU)	12.0 (7)	3.3 (−0.8 to 7.6)	↑*	40.1
Iceland (ISL)	10.1 (7)	−1.4 (−28.8 to 36.5)	↔	0.1
Lithuania (LTU)	2.0 (5)	12.3 (−34.3 to 92.1)	↔	1.6
Luxembourg (LUX)	12.7 (7)	5.4 (−11.0 to 24.8)	↔	0.2
Netherlands (NLD)	30.0 (9)	0.0 (−1.5 to 1.6)	↔	7.9
Norway (NOR)	12.7 (9)	−2.7 (−7.5 to 2.3)	↔	2.2
Sweden (SWE)	12.8 (6)	3.5 (−2.0 to 9.2)	↔	4.4
United Kingdom (GBR)	31.1 (4)	0.5 (−4.0 to 5.3)	↔	29.1
Americas excluding South America				
Canada (CAN)	10.3 (4)	5.6 (−7.4 to 20.4)	↔	15.1
Cuba (CUB)	0.6 (4)	5.2 (−36.1 to 73.2)	↔	5.6
Mexico (MEX)	2.2 (6)	2.9 (−7.2 to 14.2)	↔	49.4
United States of America (USA)	9.0 (4)	0.8 (−2.4 to 4.1)	↔	135.1
South America				
Argentina (ARG)	2.5 (7)	8.9 (3.3 to 14.7)	↑**	18.6
Brazil (BRA)	0.5 (6)	9.0 (0.1 to 18.7)	↑**	87.3
Chile (CHL)	3.1 (7)	3.3 (−8.1 to 16.2)	↔	7.5
Ecuador (ECU)	0.5 (4)	16.4 (−37.5 to 116.7)	↔	6.3
Uruguay (URY)	2.3 (5)	13.6 (−43.7 to 129.2)	↔	1.6
Oceania				
Australia (AUS)	25.5 (6)	4.6 (−0.6 to 10.1)	↑*	9.5
New Zealand (NZL)	20.5 (4)	10.4 (−10.3 to 35.7)	↔	1.9

a See “Materials and Methods” for our definition of mesothelioma.

b Period MR from 1996 to 2005, age-adjusted to the world population of 2000.

c Number of years with available data.

d APC, together with its 95% CI and *p*-values, were calculated with Joinpoint software.

e Trend: ↑ when APC > 0 (*p* < 0.10); ↓ when APC < 0 (*p* < 0.10); ↔ when *p* > 0.10 for APC.

f Average of male national population from 1996 to 2005.

* Marginally significant (0.05 < *p* < 0.10).

** Statistically significant (*p* < 0.05).

**Table 2:** Historical trend in per capita asbestos use and status of national ban.

	Use of asbestos^a^ (kg per capita/year)							
Country code	1950s	1960s	1970s	1980s	1990s	2000s	Change in use (Δ) from 1970 to 1985^b^	National ban^c^
Asia								
ISR	3.13	2.87	1.23	0.78	0.44	0.02	−0.59	No ban
JPN	0.56	2.02	2.92	2.66	1.81	0.46	0.12	2004
Others^d^ (*n* = 39)	0.06	0.15	0.25	0.27	0.30	0.31	0.05	3/39
Eastern Europe and Southern Europe								
HRV	0.39	1.13	2.56	2.36	0.95	0.65	0.49	No ban
CZE	1.62	2.36	2.91	2.73	1.30	0.14	0.21	2005
HUN	0.76	1.23	2.87	3.29	1.50	0.16	1.32	2005
POL	0.36	1.24	2.36	2.09	1.05	0.01	−0.11	1997
ROU	NA1	NA1	1.08	0.19	0.52	0.55	−1.73	2007
ESP	0.32	1.37	2.23	1.26	0.80	0.18	−1.07	2002
Others^d^ (*n* = 15)	0.79	1.57	2.35	2.05	2.35	1.72	0.30	5/15
Northern Europe and Western Europe								
AUT	1.16	3.19	3.92	2.08	0.36	0.00	−1.77	1990
DNK	3.07	4.80	4.42	1.62	0.09	NA2	−2.96	1986
FIN	2.16	2.26	1.89	0.78	NA1	0	−1.53	1992
FRA	1.38	2.41	2.64	1.53	0.73	0.00	−1.06	1996
DEU	1.84	2.60	4.44	2.43	0.10	0.00	−0.30	1993
ISL	0.21	2.62	1.70	0.02	0	0.00	−2.52	1983
LTU	NA1	NA1	NA1	NA1	0.54	0.06	NA1	2005
LUX	4.02	5.54	5.30	3.23	1.61	0.00	−2.04	2002
NLD	1.29	1.70	1.82	0.72	0.21	0.00	−1.20	1994
NOR	1.38	2.00	1.16	0.03	0	0.00	−1.72	1984
SWE	1.85	2.30	1.44	0.11	0.04	NA2	−1.96	1986
GBR	2.62	2.90	2.27	0.87	0.18	0.00	−1.41	1999
Others^d^ (*n* = 5)	3.05	4.32	4.05	2.40	0.93	0.05	−1.30	5/5
Americas excluding South America								
CAN	2.76	3.46	4.37	2.74	1.96	0.32	−1.66	No ban
CUB	NA1	NA1	NA1	0.15	0.36	0.74	NA1	No ban
MEX	0.28	0.57	0.97	0.77	0.39	0.26	0.04	No ban
USA	3.82	3.32	2.40	0.77	0.08	0.01	−1.73	No ban
Others^d^ (*n* = 12)	0.06	0.22	0.44	0.29	0.07	0.07	−0.08	0/12
South America								
ARG	NA1	0.88	0.76	0.40	0.18	0.04	−0.26	2001
BRA	0.27	0.38	0.99	1.25	1.07	0.74	0.66	2001
CHL	0.07	0.92	0.56	0.64	0.55	0.03	0.14	2001
ECU	NA1	NA1	0.67	0.52	0.14	0.26	0.29	No ban
URY	NA1	0.74	0.75	0.54	0.47	0.08	−0.20	2002
Others^d^ (*n* = 6)	0.27	0.43	0.60	0.47	0.29	0.19	−0.04	0/6
Oceania								
AUS	3.24	4.84	5.11	1.82	0.09	0.03	−2.71	2003
NZL	2.05	2.56	2.90	1.00	NA1	NA1	−2.56	No ban
Others^d^ (*n* = 3)	NA1	NA1	NA1	NA1	NA1	0.22	NA1	0/3

Abbreviations: NA 1, data not available; NA 2, not applicable because of negative use data: 0.00 when the calculated data were < 0.005; 0 if there are no data after the year the ban was introduced. See Table 1 for country codes.

a Numbers corresponding to use of asbestos by country and region were calculated as annual use per capita averaged over the respective decade.

b Change in use (Δ, kilograms per capita/year) during the period defined as the difference between the average of consumption during the former subperiod (1970–1975) and latter subperiod (1980–1985).

c Year first achieved or year planned to achieve ban. When shown as fraction, the numerator is the number of countries that achieved bans and the denominator is the number of other countries in the region.

d Data on asbestos use were available (but mortality data unavailable) for others in each region, in which case data were aggregated.

**Table 3:** Relation between recent change in pleural mesothelioma mortality and historical change in use of asbestos based on regression analyses.^a^

Period for use of asbestos	No. of countries	Adjusted *R*^2^	*p*-Value
1950			
1960	23	−0.035	0.615
1970	24	−0.038	0.689
1975	25	0.000	0.325
1980	25	0.073	0.102
1985	25	0.182	0.019
1990	27	0.277	0.003
1960			
1970	24	−0.044	0.857
1975	23	0.052	0.151
1980	27	0.201	0.011
1985	27	0.300	0.002
1990	29	0.415	< 0.001
1970			
1975	26	0.121	0.046
1980	26	0.348	0.001
1985	29	0.466	< 0.001
1990	29	0.366	< 0.001
1975			
1980	27	0.328	0.001
1985	28	0.267	0.003
1990	29	0.091	0.062
1980			
1985	28	−0.031	0.675
1990	26	−0.006	0.368
1985			
1990	27	0.037	0.170

a APC of the age-adjusted annual MRs from 1996 to 2005 (dependent variable) versus change in use during the corresponding period (independent variable).
